# Dissolved Algal Toxins along the Southern Coast of British Columbia Canada

**DOI:** 10.3390/toxins15060395

**Published:** 2023-06-13

**Authors:** Ryan B. Shartau, Lenora D. M. Turcotte, Julia C. Bradshaw, Andrew R. S. Ross, Blair D. Surridge, Nina Nemcek, Stewart C. Johnson

**Affiliations:** 1 Department of Biology, The University of Texas at Tyler, Tyler, TX 75799, USA; 2 Pacific Biological Station, Fisheries and Oceans Canada, Nanaimo, BC V9T 6N7, Canada; lenora.turcotte@dfo-mpo.gc.ca (L.D.M.T.); julia.bradshaw@dfo-mpo.gc.ca (J.C.B.); stewart.johnson@dfo-mpo.gc.ca (S.C.J.); 3 Institute of Ocean Sciences, Fisheries and Oceans Canada, Sidney, BC V8L 4B2, Canada; andrew.ross@dfo-mpo.gc.ca (A.R.S.R.); nina.nemcek@dfo-mpo.gc.ca (N.N.); 4 M.B. Laboratories, Sidney, BC V8L 5Y1, Canada; surridgeb@camosun.ca

**Keywords:** microcystin, okadaic acid, domoic acid, SPATT, harmful algae, aquaculture, marine phycotoxins

## Abstract

Harmful algal blooms (HABs) in coastal British Columbia (BC), Canada, negatively impact the salmon aquaculture industry. One disease of interest to salmon aquaculture is Net Pen Liver Disease (NPLD), which induces severe liver damage and is believed to be caused by the exposure to microcystins (MCs). To address the lack of information about algal toxins in BC marine environments and the risk they pose, this study investigated the presence of MCs and other toxins at aquaculture sites. Sampling was carried out using discrete water samples and Solid Phase Adsorption Toxin Tracking (SPATT) samplers from 2017–2019. All 283 SPATT samples and all 81 water samples tested positive for MCs. Testing for okadaic acid (OA) and domoic acid (DA) occurred in 66 and 43 samples, respectively, and all samples were positive for the toxin tested. Testing for dinophysistoxin-1 (DTX-1) (20 samples), pectenotoxin-2 (PTX-2) (20 samples), and yessotoxin (YTX) (17 samples) revealed that all samples were positive for the tested toxins. This study revealed the presence of multiple co-occurring toxins in BC’s coastal waters and the levels detected in this study were below the regulatory limits for health and recreational use. This study expands our limited knowledge of algal toxins in coastal BC and shows that further studies are needed to understand the risks they pose to marine fisheries and ecosystems.

## 1. Introduction

In British Columbia (BC), harmful algal blooms (HABs) have a significant impact on the salmon aquaculture industry, with direct losses due to HABs exceeding $16 million Canadian dollars from 2009–2012, and indirect costs, such as reductions in growth and costs of monitoring and mitigation, ranging from $4 to 8 million Canadian dollars annually [1]. Salmon are typically farmed in areas frequented by wild salmon, and thus, algal toxins also represent a potential threat to wild salmon. Salmon are an integral part of the trophic food web, including a food source for endangered and threatened resident orcas [2]; thus, algal toxins, to which they are exposed, may have far reaching impacts on the ecosystem’s health.

The algae most frequently associated with such impacts are *Chaetoceros* spp., which cause mechanical damage of gills, and *Heterosigma akashiwo*, for which the mode of toxicity is not yet understood [3]. However, there are other diseases that can have large impacts on the production of farmed fish, and which are believed to be due to the exposure to algal toxins [4]. These include diseases caused by exposure to the silicoflagellates *Octactis speculum* and *Dictyocha fibula* which, based on the signs of disease and histopathology, are considered ichthyotoxic [1,3,5]. Another example is Net Pen Liver Disease (NPLD), which is characterized by a loss of gross liver structure, histological changes (including diffuse necrosis, vacuolation, and megalocytosis of the liver parenchyma) and, in some cases, high levels of morbidity [6,7]. There is evidence that the occurrence of NPLD at Atlantic Salmon farms in the Northeastern Pacific has increased in frequency over the last several years [6].

Documented HABs impacts on wild salmon include the reduction of feeding activities during blooms, mechanical damage of gills by *Chaetoceros* spp., and ichthyotoxic effects of *Octactis speculum* on Chinook salmon (*Oncorhynchus tshawytscha*), as evidenced by gross and histopathological signs [5]. With respect to NPLD, the presence of hepatic megalocytosis has also been reported from farmed Steelhead Trout (*O. mykiss*), wild and farmed Chinook Salmon, and wild Pink Salmon (*O. gorbuscha*) sampled in marine waters of the Northeastern Pacific [6,8,9]. NPLD is believed to be caused by the exposure to microcystins (MCs), a well-recognized class of potent liver toxins [7,10]. Although most commonly reported in freshwater systems [11,12,13,14,15], MCs have been detected in marine environments worldwide, including in California [16,17,18,19,20], Eastern United States [21,22,23], Japan [24], and Greenland [25].

MCs are well-studied cyanotoxins (e.g., [14,26,27]) with over 270 congeners [28,29], and are produced by numerous genera of cyanobacteria, including *Anabaena* (*Dolichospermum*), *Aphanizomenon*, *Planktothrix* [30], and commonly by *Microcystis* [31]. They are potent hepatotoxins that are linked to changes in gene expression, physiology, and morphology in numerous animals, including fish [4,32,33,34,35,36], invertebrates [36,37,38,39,40], as well as marine and terrestrial mammals [20,41]. In addition to causing severe liver damage, they can induce a variety of sub-lethal effects in fish [42], which include cardiorespiratory function [43,44], reproductive and endocrine function [45,46,47,48], growth rate [49], whole animal condition [50], swimming performance [51], and immunity [52,53,54]. Microcystins exert their toxic effects via the inhibition of protein phosphatases (PP1 and PP2A), which affect the regulation of the cellular protein phosphorylation, and result in disturbances in cellular phosphorylation homeostasis that may lead to cytogenetic and turmorigenic effects, or the promotion of hepatocyte necrosis [55].

In addition to MCs, okadaic acid (OA) and analogs (Dinophysistoxins or DTXs) are also potent hepatotoxins associated with blooms of *Dinophysis* in BC [56] and along the west coast of North America [57]. The mechanism of OA toxicity is similar to MC, as it also inhibits protein phosphorylation, and sub-lethal exposure induces neurotoxic, immunotoxic, and genotoxic effects due to its inhibition of protein phosphatases and oxidative stress in non-salmonid fishes [58]. Histopathological changes in marine fish associated with OA exposure are reported in gill (hypertrophy and fusion of the primary lamellae) and liver tissues (hyperemia, vascular dilation, hepatocyte size, and membrane disintegration) [59,60]. Dissolved OA was found to induce severe mortality in Longfin yellowtail *Seriola rivoliana* embryos [61].

There are different routes by which marine fish may be exposed to toxins, such as the direct uptake of phytoplankton or dissolved toxins, by ingestion or adsorption across gills or other surfaces, and/or indirect exposure through the consumption of contaminated feeds [62]. In situations where fish are exposed to waters above the isosmotic salinity, various osmoregulatory mechanisms are employed, including high rates of drinking (reviewed in [63]). For example, drinking rates in seawater-acclimated Rainbow Trout, Coho Salmon, and Atlantic Salmon have been measured to be up to 129, 288, and 192 mL/h/kg, respectively (reviewed in [64]). For all life history stages of estuarine and marine fish, drinking for the purpose of osmoregulation results in chronic exposure to dissolved and particulate associated algal toxins through ingestion. The chronic sublethal and lethal effects of such exposure in marine fish as a result of drinking are unclear.

As part of a study into the occurrence of NPLD, and given the lack of information about algal toxins in BC marine environments and the potential risk they pose to marine ecosystems, our objective was to investigate the presence of MCs at sites important to salmon aquaculture, along with other algal toxins that are a known threat to aquaculture, including OA, domoic acid (DA), dinophysistoxin-1 (DTX-1), pectenotoxin-2 (PTX-2), and yessotoxin (YTX). Sampling occurred over a two year period by measuring toxin concentrations in discrete water samples and Solid Phase Adsorption Toxin Tracking (SPATT). SPATT is a passive sampling method that has been used in field studies to examine a range of phycotoxins, including MC, diarrhetic shellfish poisoning (DSP) toxins (e.g., OA, DTX-1), DA, and YTX [23,65,66]. The use of SPATT provides greater temporal resolution and increased toxin detection capabilities (e.g., higher sensitivity and ability to detect multiple toxins) relative to discrete water samples [67,68]. Samples were measured at several sites adjacent to salmon farms in the Strait of Georgia (SOG) (located between Vancouver Island and the BC mainland) and along the west coast of Vancouver Island (WCVI), BC. As this region contains over a hundred farms (https://www.dfo-mpo.gc.ca/aquaculture/bc-cb/maps-cartes-eng.html; accessed on 12 May 2023), is in proximity to several major salmon runs—including the Fraser River [69]—and is home to approximately 3.4 million people, it is an important marine environment in which to identify the presence and persistence of algal toxins. This study represents the first concerted effort to measure algal toxins in coastal BC.

## 2. Results

### 2.1. Toxins Detected in Solid Phase Adsorption Toxin Tracking (SPATT)

**Figure 1:**
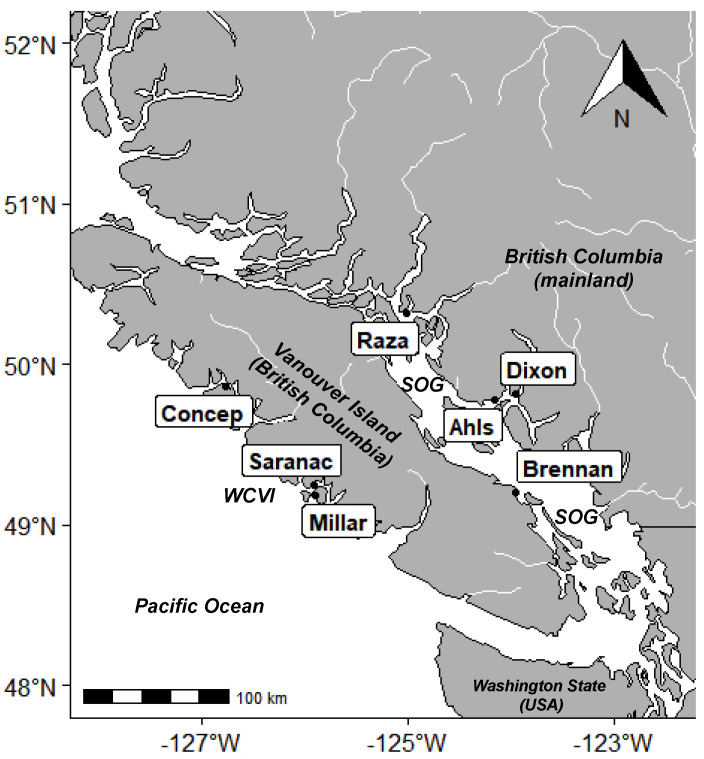
Location of sampling sites in British Columbia, Canada. Outlined labels indicate farm sites: Concepcion (Concep.), Saranac Island (Saranac), Millar Island (Millar), Raza Island (Raza), Ahlstrom (Ahls), Dixon Bay (Dixon), and Brennan Island (Brennan). SOG—Strait of Georgia, WCVI—west coast Vancouver Island.

**Figure 2:**
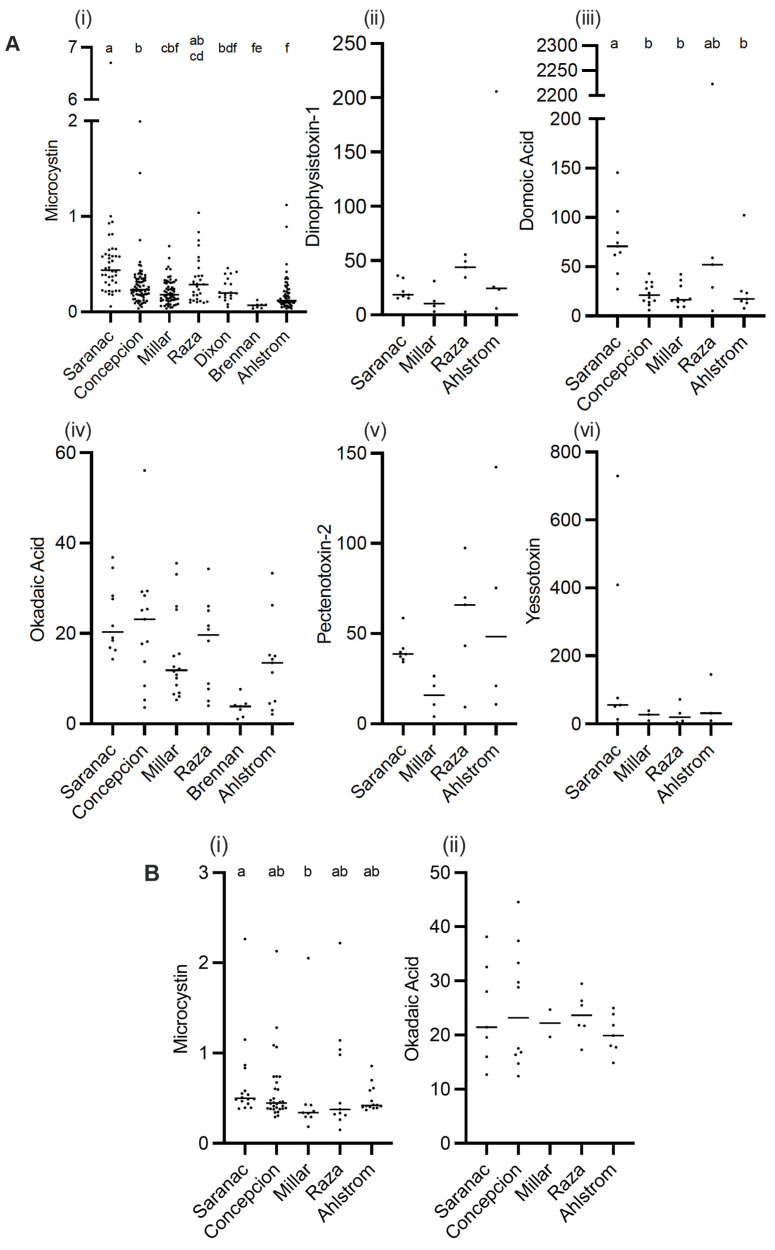
Toxin concentrations for each site over the time period collected for SPATT (**A**) and discrete water samples (**B**). SPATT (**A**): microcystin (MC; ng MC/g resin (dry weight)/day) (i); domoic acid (DA; pg DA/g resin dry weight/day) (ii); dinophysistoxin-1 (DTX-1; ng DTX-1/g resin dry weight/day) (iii); okadaic acid (OA; ng OA/g resin dry weight/day) (iv); pectenotoxin-2 (PTX-2; ng PTX-2/g resin dry weight/day) (v); yessotoxin (YTX; ng YTX/g resin dry weight/day) (vi). Discrete water: MC (ng MC/L) (i); OA (ng OA/L) (ii). Data are mean ± SEM; different letters indicate significant differences (*p* < 0.05) in toxin concentration between sites; see text for details.

**Figure 3:**
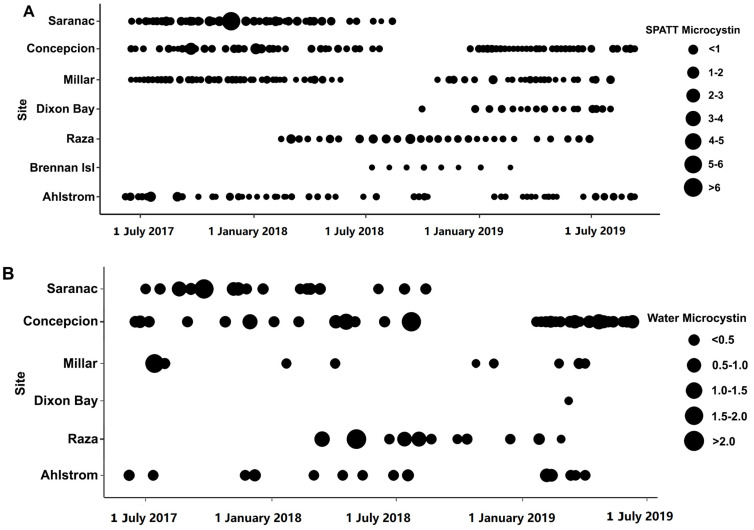
Microcystin (MC) concentration in SPATT (ng MC/g resin dry weight/day) (**A**) and water samples (ng MC/L) (**B**) from June 2017–September 2019 at sites along the west coast of Vancouver Island and the Strait of Georgia in British Columbia.

**Figure 4:**
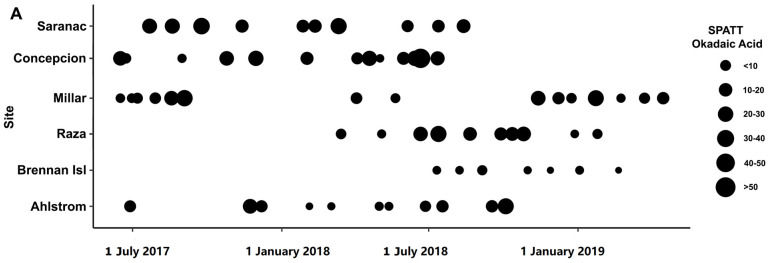

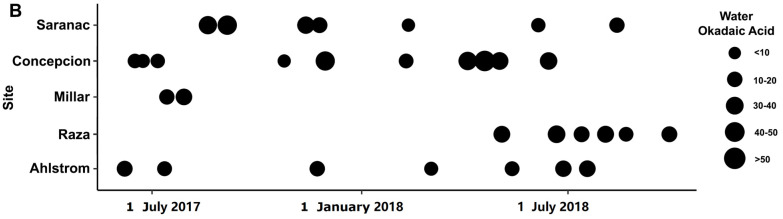
Okadaic acid (OA) concentration in SPATT (ng OA/g resin dry weight/day) from June 2017–April 2019 (**A**) and water samples (ng OA/L) from June 2017–September 2018 (**B**) at sites along the west coast of Vancouver Island and the Strait of Georgia in British Columbia.

**Figure 5:**
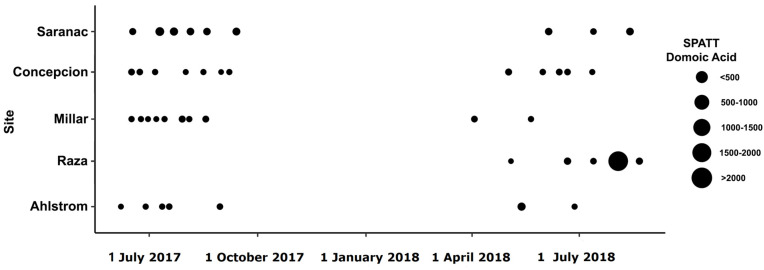
Domoic acid (DA) concentration in SPATT (pg DA/g resin dry weight/day) from June 2017–August 2018 at sites along the west coast of Vancouver Island and the Strait of Georgia in British Columbia.

Toxins were detected in all SPATT samples from all seven sampling sites (Figure 1). Microcystins were the toxin of primary interest in this study, and thus, the most extensive measurements were collected for this toxin. In all SPATT samples (n = 283), MCs were detected with concentrations ranging from 0.037 to 6.704 ng MC/g resin dry weight/day across all sites (Figure 2A and Figure 3A; Table 1). The Kruskal–Wallis test revealed significant differences in MC concentrations across sites (H = 76.2 (7), *p* < 0.0001; Figure 2A). Similarly, in all SPATT samples measured, OA and DA were detected, with concentrations ranging from 1.09 to 56.06 ng OA/g resin dry weight/day (n = 66) (Figure 2A and Figure 4A; Table 2) and 5.1 to 2223.0 pg DA/g resin dry weight/day (n = 43) across all sites (Figure 2A and Figure 5; Table 3), respectively. There were differences in DA (H = 16.6 (5), *p* < 0.05; Figure 2A), but not OA, concentrations across sites (H = 8.9 (5), *p* = 0.06; Figure 2A).

**Figure 6:**
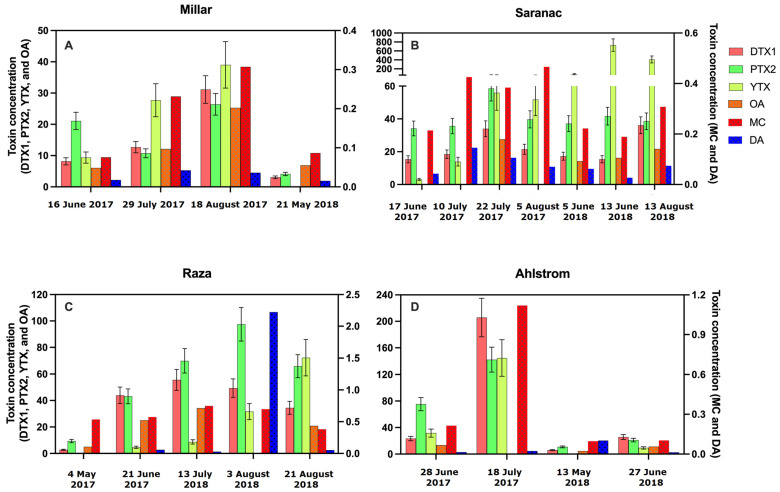
Concentrations of co-occurring toxins found in SPATT at Miller (**A**), Saranac (**B**), Raza (**C**), and Ahlstrom (**D**) at selected dates during 2017 and 2018 (Day Month Year). Dinophysistoxin-1 (DTX-1; ng DTX-1/g resin dry weight/day), pectenotoxin-2 (PTX-2; ng PTX-2/g resin dry weight/day), and yessotoxin (YTX; ng YTX/g resin dry weight/day) were measured using LC-MS/MS. Reproducibility was evaluated for LC-MS/MS by running a test sample 7 times, which yielded relative standard deviation (RSD) values of 14.2, 13.1 and 19.0%, respectively, for DTX1, PTX2, and YTX; standard deviation for each sample was calculated using RSD to produce error bars for these toxins. Okadaic acid (OA; ng OA/g resin dry weight/day), microcystin (MC; ng MC/g resin dry weight/day), and domoic acid (DA; ng DA/g resin dry weight/day) were measured using ELISA; error bars could not be calculated for individual samples measured using ELISA.

The LC-MS/MS analysis of SPATT extracts from selected sites revealed the presence of DTX-1, PTX-2, and YTX co-occurring in all samples measured (Figure 2A and Figure 6; Table 4, Table 5 and Table 6). DTX-1 ranged from 2.8 to 205.8 ng DTX-1/g resin dry weight/day (n = 20), PTX-2 ranged from 4.1 to 142.3 ng PTX-2/g resin dry weight/day (n = 20), and YTX ranged from 3.1 to 729.7 ng YTX/g resin dry weight/day (n = 17). No differences in these toxins occurred across sites (DTX-1 − H = 4.2 (4), *p* > 0.05; PTX-2 − H = 6.3 (4), *p* > 0.05; YTX − 2.4 (4), *p* > 0.05). These toxins (DTX-1, PTX-2, YTX) co-occurred in samples where MC, OA, and DA were detected using ELISA (Figure 6).

When considering regional differences between samples collected off the WCVI to those collected at sites within the SOG, concentrations of MCs detected in SPATT were significantly higher (Mann–Whitney *U* = 6737, n_1_ = 173, n_2_ = 110, *p* < 0.0001 two-tailed) for the SOG (median = 0.24 ng MC/g resin dry weight/day; n = 173) compared to the WCVI (median = 0.16 ng MC/g resin dry weight/day; n = 110). Similarly, the OA concentration in SPATT samples was significantly higher (*t* = (65) 2.741, *p* < 0.01) at sites in the SOG (median = 17.68 ng OA/g resin dry weight/day; n = 39) compared to sites on the WCVI (median = 8.331 ng OA/g resin dry weight/day; n = 28). No significant differences in the concentration between these regions were observed for DA, DTX-1, PTX-2, or YTX in SPATT samples.

### 2.2. Toxins Detected in Grab Samples

**Figure 7:**
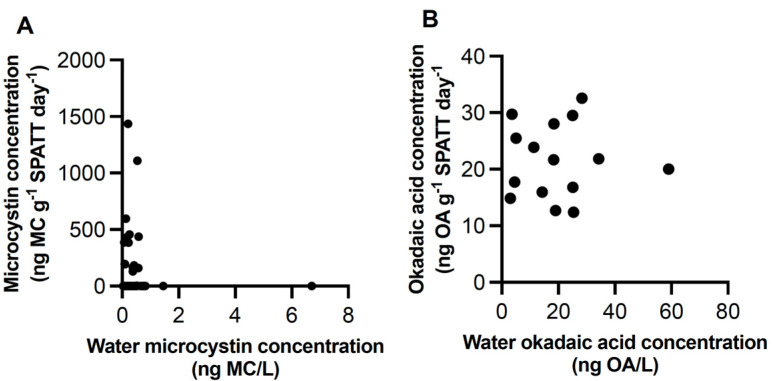
Correlation of microcystin (**A**) and okadaic acid (**B**) in SPATT and water samples. SPATT values were ng toxin/g of dry weight of SPATT resin/ day of deployment time; these were compared to toxin concentration in water (ng toxin/L) collected at the same time SPATT bags were collected.

Discrete water samples were examined at selected sites for MC and OA. In all samples, both toxins were detected. MC concentrations ranged from 0.1477 to 2.265 ng/L (n = 81) (Figure 2B and Figure 3B; Table 1). OA concentrations ranged from 12.41 to 44.56 ng/L (n = 32) (Figure 2B and Figure 4B; Table 2). The concentration of MC (H = 9.933 (5), *p* < 0.05), but not OA, in water samples differed across sites; no differences occurred between the WCVI and SOG for either toxin. The concentrations of MCs (Figure 7A) and OA (Figure 7B) in water samples were not correlated with those detected in the SPATT samples.

## 3. Discussion

This study, using SPATT and water samples, was the first to show that microcystins and other phycotoxins co-occur throughout the nearshore waters of Vancouver Island and Southern BC mainland coast. Microcystins were the primary toxin of interest and were detected over a two year period at four sites in the SOG and three sites on the WCVI. In addition, three other toxins were detected in SPATT samples: DTX-1, PTX-2, and YTX. 

Toxin concentrations obtained by water and SPATT were not directly comparable and no correlation between the two methods was observed for MCs and OA in this study (Figure 7). Discrete water samples are generally believed to underestimate the presence of toxins compared to SPATT, but it has been suggested that there is approximately a correspondence of 10:1 for SPATT to water samples [66,67]. Despite the differences between SPATT and water toxin concentrations, and although the amount of toxins detected in such samples cannot be directly related to environmental concentrations without validating uptake rates, interactions and degradation of toxins in SPATT samplers, the use of normalized data (e.g., ng toxin/g SPATT resin/day) makes it possible to compare relative amounts across the region over time [23,66,70]. Detection of these toxins provided a starting point for further investigations into their temporal and spatial distribution along the coast of BC and builds on existing studies documenting phycotoxins in other coastal regions [3,14,16,17,18,21,23].

SPATT extracts tested positive for multiple toxins (Figure 2A, Figure 3A, Figure 4A and Figure 5), showing that they co-occur in time and space. Of the 283 SPATT extracts analyzed for MC, 48 were analyzed for either DA or OA, and 27 for both DA and OA, all of which were found to contain these toxins. Some extracts were also analyzed using LC-MS/MS, which indicated that 17 extracts had 6 co-occurring toxins and 1 extract had 3 co-occurring toxins (Figure 6).

The amount of toxins in extracts did not vary greatly between sites or by season, with MCs present in relatively stable amounts over the seasons and between sites. Spatially, sites on the WCVI had higher amounts of MCs and OA than sites in the SOG; DA did not differ between those regions. However, gaps in sampling were present because some of the farms at which SPATT samplers were deployed by staff were not operated continuously year-round, limiting our ability to infer seasonal changes at these sites. There was insufficient data to comment on the spatio–temporal distributions of DTX-1, DTX-2, and YTX; however, there was an increase in YTX during Summer 2018 at the Saranac Island site on the WCVI (Figure 6).

### 3.1. Microcystin

Microcystins were found in SPATT and water extracts at all sites; however, because the ELISA kit cannot differentiate between congeners, the reported MC concentrations represent the total abundance of all MC congeners detected using the kit. It was likely that the majority of these are MC–LR or MC–RR, which are considered to be the most prevalent congeners in aquatic systems [14,28,29]. In water and SPATT samples collected in California, the dominant was MC–RR, which occurred in 91% of both samples, followed by MC–LR (78 and 82%, respectively) [17]. In contrast, on the east coast in Chesapeake Bay, detection of MCs occurred at only one site where MC–LR, MC–RR, and MC–YR were included in the analysis, but only MC–LR was detected [23]. The concentrations of MC detected in SPATT in this study were generally low compared to other studies using comparable methods. Our samples ranged from 0.07 to 0.62 ng MC/g resin dry weight/day, with a maximum of 6.7 ng MC/g resin dry weight/day (Figure 2A; Table 1), whereas studies in California recorded 1.5 to 2.8 ng MC/g resin dry weight/day [17]. Similarly, MC concentrations of 0.48 to 0.69 ng/L (Figure 2B; Table 1) in water were lower than levels documented to occur in San Francisco Bay (1.2 to 18.5 ng/L) [17], Chesapeake Bay (658 ug/L) [22], and other marine locations worldwide [16].

Although the detection of MCs in SPATT and water extracts from these marine sites was a novel finding, the concentrations were still far below the regulatory limits for health and recreational use. The United States Environmental Protection Agency health advisory limit in freshwater for MCs is 0.3 ug/L (US EPA 2019), the World Health Organization lifetime and short-term drinking water values are 1 and 12 µg/L [17], respectively, and the Health Canada guideline for total microcystins in recreational waters used for primary contact recreation is a maximum concentration of 10 µg/L [71]. This suggests that MCs in these areas of coastal BC at the locations and times measured were below the recognized levels of environmental or health risks.

### 3.2. Okadaic Acid

Okadaic acid was detected in all SPATT and water extracts from all sites measured (Figure 4). Concentrations of OA in SPATT ranged from 1.087 to 56.06 ng OA/g resin dry weight/day (Figure 2A; Table 2), which were lower than levels detected in SPATT samples from Southern California [18], but comparable to those in Chesapeake Bay and Virginia coastal bays [23]. Water concentrations ranged from 12.41 to 44.56 µg/L (Figure 4B; Table 2); however, since OA is easily accumulated by shellfish and finfish, and subsequently consumed by humans, resulting in diarrhetic shellfish poisoning (DSP), most studies measure OA concentrations in mussel tissue for which the regulatory guideline is 160 µg/kg [17,58,72]. It was unclear whether the levels of OA measured in SPATT and water samples during this study represent a threat to wildlife and humans. However, in BC, the threat posed by diarrhetic shellfish toxin (DST), which include OA, has resulted in the periodic closure of shellfish harvesting areas [3]. Dinoflagellates of the genus *Alexandrium*, which produce OA, have been observed to be abundant in BC’s Strait of Georgia (SOG) from 2015 to 2017. These organisms, which were most common in shallower nearshore regions, but did not result in heavy blooms, were found to be negatively affected by the 2015 El Niño conditions and positively by the 2017 La Niña conditions [73], indicating OA levels may vary greatly depending on climatic changes. Species of *Dinophysis*, another potential source of OA, were estimated to have increased in surface abundance by up to 10% between 2017 and 2019 in SOG surface waters, where 4 of our sampling sites were located, and by up to >21% in the region near the Ahlstrom site (https://maps.sogdatacentre.ca/documents/psfsogdc::harmful-algae-map-document-series-sog-dinophysis-abundance-for-2015-to-2019/explore; accessed on 12 May 2023).

### 3.3. Domoic Acid

As with MCs and OA, DA was detected in all SPATT extracts (Figure 5), ranging from 5 to 2223 pg DA/g resin dry weight/day (Figure 2A; Table 3), which was lower than the maximum levels detected in California [17,18] by an order of magnitude. Although low compared to California, the maximum DA levels in this study were still higher than the maximum DA in Chesapeake Bay and Virginia coastal bays [23]. In the SOG, BC blooms of *Pseudo-nitzschia*, the DA-producing algae, were recorded only once in the Northern region of the SOG between 2015 and 2018—DA is not generally a concern in this region [73]. Another monitoring program that ran from 2014 to 2017 in Cowichan Bay, BC, only detected two *Pseudo-nitzschia* blooms during this period, both in 2014 [5]. Recent monitoring of Canada’s Pacific marine waters near Vancouver Island for DA from 2016 to 2021 found the most common concentration range for samples containing DA was greater than 1 and less than or equal to 10 pg/mL (21%) (R.I. Perry, pers. Comm.). Concentrations of DA over 100 pg/mL were deemed ‘concentrations of concern’, and only occurred in 4.6% of samples. In samples where DA was detected (i.e., zero values excluded), the mean DA concentration during the 2016–2021 study period was 28.4 pg/mL, with a median of 0.82 pg/mL and DA concentrations were significantly higher on the WCVI than in the SOG (R.I. Perry, pers. Comm.), unlike in this study. It is important to recognize that levels of DA may be underestimated by SPATT, as DA is a hydrophilic compound and susceptible to loss during water rinses prior to extraction from resin [68].

### 3.4. DTX-1, PTX-2, and YTX

These three toxins were measured using LC–MS/MS at selected sites and times. DTX-1 and PTX-2 were detected in all SPATT extracts analyzed by LC–MS/MS, ranging from 2 to 205 and 4 to 142 ng toxin/g resin dry weight/day, respectively (Figure 2A; Table 4, Table 5 and Table 6). These values were greater than those detected in Chesapeake Bay and Virginia coastal bays [23], and their persistent year-round presence could be due to low background cell abundances of the causative organism, *Dinophysis* spp., similar to what was observed in Chesapeake Bay [23]. YTX was detected in all 17 samples analyzed, ranging from 3 to 729 ng YTX/g resin dry weight/day. The only other study, of which we are aware, that involved the measurement of YTX in SPATT extracts was carried out in Chesapeake Bay and Virginia coastal bays; however, YTX was not detected at any sites or time points [23]. However, YTX has been identified in contaminated shellfish worldwide [74,75,76], including as the cause of a mass mortality event of abalone in California [77].

### 3.5. Co-Occurrence of Toxins and Distributions

Multiple toxins were found to co-occur during this study. Where measured, multiple toxins were detected in all extracts (Figure 6), with 17 extracts containing 6 toxins. Co-occurrence of multiple toxins had also been observed in California [17,18], Texas [78], Chesapeake Bay and Virginia coastal bays [23], Bogue Sound North Carolina [21], as well as in freshwater systems [15]. The co-occurrence of toxins likely reflects the presence of multiple harmful algae species; in the Chesapeake Bay, at least 37 species of harmful algae have been documented to co-occur [79]. Several harmful algal species have also been documented at the same time on the WCVI and have likely been responsible for fish-killing events [1], with 14 known harmful species occurring in Barkley Sound during surveys over a 1 year period [56].

No obvious trends in spatial or temporal distribution were observed for the toxins measured in this study. This may have been due to the limited sampling resolution and the fact that sampling did not occur on a regular basis at all sites. Results here likely reflect background levels within the region in the absence of blooms, but changes in climate, along with exceptional climate anomalies—including those previously observed in the Northeast Pacific Ocean [57,80,81,82]—may result in larger harmful algal blooms. However, it is interesting to note that the WCVI had higher MC and OA levels than the SOG, suggesting that the former is more favorable for the formation of associated harmful algal blooms. This would be worth investigating further as differences in toxins may influence management practices for aquaculture and fisheries.

The presence of MC in all samples suggests that these toxins are being continually produced, despite the absence of large cyanobacterial blooms (none of which were reported during the study period at the sites investigated). This may be a consequence of their stability, as MCs (and certain other toxins) can persist for weeks to years, depending on the environmental conditions [83,84,85,86,87]—meaning they could still be present long after blooms occur. Additionally, the apparent absence of MC-producing blooms in the marine environment may indicate that the MCs detected here have freshwater origins; downstream transport of MCs have been suggested to be a source of dissolved MCs in the Bogue Sound North Carolina [21]. Freshwater *Microcystis aeruginosa* blooms are recognized as a source of marine toxins [16,17,27]. The region where sampling occurred in this study is fed by numerous freshwater systems, although the presence of harmful algal blooms in those waters is not known.

Irrespective of their origins, these algal toxins are likely to have some effect on marine organisms. Given their association with NPLD in farmed and wild salmon [10], MCs are likely responsible for severe mortality events at fish farms [7]. A recent study exposing Atlantic and Chinook Salmon to a single oral dose of MCs did not result in NPLD, but produced hepatic lesions [64], along with changes in gene expression associated with immune and inflammatory responses (Shartau, unpublished). Results here and elsewhere suggest that fish are likely to be exposed to multiple toxins simultaneously. This may result in additive or synergistic effects that exacerbate toxic responses following exposures, and may contribute to incidences of mortality and morbidity observed in wild animals [8,20,21,78]; however, it is not known what toxins concentrations elicit potential toxic responses.

Salmon and other marine fishes drink seawater at a higher rate than freshwater fish [88,89], and would likely be exposed continuously to toxins present in the environment. In freshwater Rainbow Trout, exposure to toxins may increase drinking rate, resulting in an osmoregulatory imbalance due to the increased fluid in the gut and inability to remove the excess water [89]. It is unclear how continuous exposure via drinking impacts marine fishes; however, drinking results in cumulative exposure to increasing toxin levels, as seen in Table 7. Due to the higher drinking rate of fishes in seawater compared to freshwater, it is likely marine fishes will be exposed to greater toxin concentrations [89]. Even between salmon species, drinking rates can vary, as drinking rates in marine Coho Salmon was 0.012 L/h/kg [88], while Atlantic Salmon was up to 0.010 L/h/kg (Laronde and Brauner, unpublished). Cumulative toxin uptake at these drinking rates may have sub-lethal effects, and consequently, microcystins and other toxins may pose a greater threat to fishes in marine environments compared to freshwater; this is an area needing further investigation. Furthermore, as these monitoring efforts only cover a limited time period, it is not known if these toxin levels are part of the normal environmental background or represent higher-than-historical levels. If these toxins naturally co-occur at these levels, then it is likely marine animals have adapted to these levels and it may not represent a physiological challenge.

### 3.6. Relevance and Future Directions

The west coast of Canada is home to a large aquaculture industry for finfish and shellfish, both of which are affected by harmful algal toxins. Until now, the presence and distribution of toxins—particularly MCs—had not been investigated, so it was largely unclear as to what toxins were present and where in regions where finfish aquaculture occurs. At salmon farms, harmful algae are associated with numerous fish-kill events and likely induce sub-lethal impacts on these fish, which creates economic and management challenges for producers. As wild salmon and other marine animals are present in the same waters, algal toxins may be responsible for morbidity and mortality in these organisms. Importantly for conservation managers, some wild salmon populations have been in decline (Noakes et al., 2000). That the presence of these toxins could be a contributing factor merits further investigation.

As this was the first attempt at characterizing algal toxins along coastal BC, it would be valuable to continue monitoring for toxins of interest to establish a baseline to which future changes could be compared to. In the future, toxin measurements should be linked with the presence of harmful algae, such as the work done by Esenkulova et al. [73] to understand the relationship between blooms and toxins. The presence of multiple co-occurring toxins suggests it would be valuable to understand how each of these toxins impact marine animals, especially salmon, and how these toxins interact with each other. Another area that should be examined is investigating the rate and route of uptake in fishes, which would inform on the ecologically relevant toxin levels experienced by the individuals in these environments, including how higher drinking rates in marine fishes may impact exposure to toxins compared to those in freshwater. As blooms of harmful algae are projected to increase due to climate change and human activities [57], understanding the presence, concentration, and distribution of toxins, along with how they are taken up and impact marine fishes, will be important for the aquaculture industry, conservation agencies, and recreational users.

## 4. Materials and Methods

### 4.1. Sample Collection

Seven sites around the Southern coast of BC were sampled between June 2017 and June 2019 (Figure 1); Table 8 contains the range of dissolved O_2_, salinity, and temperature at each site. At each site, samples were taken using SPATT samplers, which were constructed based on previously used methods [67,68]. Bags were made using 100 µm Nitrex bolting cloth and filled with 3 g of Diaion HP20 resin, which was then activated by soaking in 100% methanol (MeOH) for 48 h, rinsed in Milli-Q water to remove MeOH, and then transferred to fresh de-ionized water for storage at 4 °C prior to use. At deployment sites, duplicate SPATT bags were placed inside plastic mesh cages to allow water flow and discourage colonization by animals and plants. SPATT bags were deployed at a depth of 1 m for approximately 1 week and deployment and retrieval dates were recorded. Once SPATT samplers were collected from the field, they were frozen at −20 °C until processing.

Toxins were recovered from SPATT samplers using a solid phase extraction (SPE) vacuum manifold. The resin was transferred from the SPATT bag to an empty 20 mL fritted column (Varian) on the manifold using a funnel and rinsed with up to 4 mL of distilled water, which was eluted to waste at 1–2 mL min^−1^ by slowly opening the manifold valve. The SPATT resin was then rinsed with another 26 mL of DI water (for a total rinse volume of 30 mL). Next, 4 mL of MeOH was added to the resin, stirred, and eluted into a 25 mL volumetric flask. Another 6 mL of MeOH was added to the resin, which was stirred, covered with parafilm, and allowed to soak for 30 min before slowly eluting the MeOH into the volumetric flask. Finally, 10 mL of MeOH was added and eluted to dryness. The volumetric flask was then removed from the vacuum manifold and the final volume of MeOH in the flask was brought up to 25 mL. This was transferred to a 40 mL glass vial, which was placed in an evaporator (Visiprep, Supelco, Inc., Bellefonte, PA, USA). Nitrogen was used to evaporate the MeOH, which occurred at 30 °C, to facilitate this process. Once MeOH was evaporated, the residue was hydrated with 1 or 2 mL of ultrapure HPLC grade water. Samples were used immediately or stored frozen at −20 °C prior to analysis.

Discrete water samples were collected at a depth of 0.5 m, adjacent to where SPATT sampling occurred, using 1 L PETG (polyethylene terephthalate glycol) bottles, which have a low binding affinity for toxins [90]. Samples were stored frozen at −20 °C, and were thawed and re-frozen 3 times prior to extraction to ensure lysis of algal cells. Two SPATT bags were then placed in each bottle and the samples were agitated daily for a period of 1 week. The bags were then removed, and the resin was extracted, as described above.

### 4.2. Toxin Analysis by ELISA

SPATT extracts were analyzed by the Enzyme-Linked Immunosorbent Assay (ELISA). For microcystins, okadaic acid, and domoic acid, the manufacturer’s guidelines were followed for each ELISA kit. Analysis for the ELISA kits used in this study were the Abraxis Microcystin-DM ELISA kit (Abraxis LLC, Warminster, PA, USA), the ASP ELISA kit for quantitative determination of domoic acid (product no: A31300401; Biosense laboratories AS, Bergen Norway), and the Abraxis Okadaic Acid ELISA kit (Abraxis LLC, Warminster, PA, USA). We followed the manufacturer’s guidelines, which used standards (e.g., for microcystins, this included standards of 0, 0.15, 0.40, 1.0, 2.0, and 5.0 ppb), blank, and control (e.g., for microcystins, this was 0.75 ppb). When running the ELISA assay, the standard curve R squared was >0.99, and the control was within the accepted range indicated by the manufacturer (e.g., for microcystin, this was 0.75 +/− 0.185 ppb).

The detection limits for all toxins in this analysis were <0.2 ppb. Consistent with other studies [15], samples were diluted using molecular water as necessary to allow monitored levels to fall within the calibration range of the individual ELISA kits. Some undiluted extracts were also analyzed by reversed-phased liquid chromatography-tandem mass spectrometry (LC–MS/MS) to provide information about hydrophobic toxins amenable to analysis using the LC–MS/MS method.

### 4.3. Toxin Analysis by LC–MS/MS

Selected SPATT extracts were analyzed by LC–MS/MS using a quadrupole tandem mass spectrometer (Xevo TQ-S; Waters Corporation, Milford, MA, USA) coupled via an electrospray ionization (ESI) interface to an ultra-high performance liquid chromatograph (Aquity H-class FTN UPLC; Waters). Reversed-phase liquid chromatography was performed using a Luna C18 analytical column (3 μm, 4.6 × 100 mm; Phenomenex), a solvent flow rate of 0.45 mL min, a column temperature of 30 °C, and an injection volume of 3 μL. Separations were carried out using a binary solvent system comprising of 4 mM ammonium formate with 0.1% *v*/*v* formic acid in methanol (Solvent A), and 4 mM ammonium formate with 0.1% *v*/*v* formic acid in water (Solvent B). The composition of the mobile phase at the start of each run was 30:70 A:B. This was held for 1 min before ramping to 60:40 A:B at 2 min and to 80:20 A:B at 3.2 min before holding until 15 min, during at which time hydrophobic biotoxins eluted from the column. The mobile phase composition was then returned to 30:70 A:B at 15.2 min and held until 18 min to recondition the column. ESI interface parameters were capillary voltage 2230 V, desolvation gas flow 750 L/h, desolvation temperature 450 °C, and source temperature 150 °C.

Pectenotoxin-2 was detected as protonated molecular (precursor) ions in the positive ion mode, and dinophysistoxin-1 and yessotoxin as deprotonated molecular ions in the negative ion mode. Multiple reaction monitoring (MRM) of the most intense (primary) precursor-to-product ion transition was used to quantify each compound, while another (secondary) transition was used to confirm its identity, with the collision energy (CE) optimized for each MRM transition (Table 9). Calibration was carried out using biotoxin certified reference materials (National Research Council of Canada, Ottawa, ON) dissolved in methanol at concentrations ranging from 0.8 to 120 ng/mL. SPATT extracts were thawed at room temperature and 200 µL of each extract pipetted into a 2 mL amber vial, to which 250 µL of 80:20:1 water/acetonitrile/acetic acid was added. Each solution was then made up with acetonitrile to a total volume of 1.2 mL, of which 3 μL was injected for analysis. Results were used to determine the concentration and amount of each biotoxin in the original 2 mL SPATT extracts, from which the amounts of biotoxin per gram of resin were subsequently determined. The detection limit for toxins in this analysis was 2 ng/g resin dry weight.

### 4.4. Statistical Analyses

Data were compared by the Welch *t* test or, where multiple treatments were evaluated, data were analyzed by an analysis of variance (ANOVA), followed by the Tukey post hoc test to compare all groups with each other. When data did not meet normality (Shapiro–Wilk normality test) or equal variance (Bartlett test) assumptions, a Mann–Whitney test or Kruskal–Wallis test, followed by a Dunn multiple comparison test, was used (*p* < 0.05) to confirm conclusions. Correlation between SPATT and water samples was performed using the non-parametric Spearman correlation. GraphPad Prism (v.9.5.1) was used for all statistical analyses. GraphPad Prism (v.9.5.1) and R (v.4.1.2) were used for the preparation of figures.

## Figures and Tables

**Table 1:** Microcystin concentrations in discrete water samples and SPATT.

Site	Water Microcystin Concentration (ng MC/L)					Microcystin Concentration in SPATT (ng MC/g SPATT Dry Weight/Day)				
	Min	Max	Median	Mean (SE)	n	Min	Max	Median	Mean (SE)	n
Ahlstrom	0.369	0.855	0.419	0.489 (0.039)	14	0.037	1.120	0.119	0.191 (0.026)	55
Concepcion	0.290	2.128	0.446	0.589 (0.069)	30	0.037	1.992	0.233	0.296 (0.033)	71
Dixon Bay	Not measured					0.053	0.406	0.199	0.239 (0.029)	18
Millar	0.185	2.050	0.340	0.522 (0.193)	9	0.038	0.689	0.181	0.213 (0.017)	61
Raza Island	0.148	2.218	0.376	0.689 (0.186)	11	0.097	1.038	0.290	0.335 (0.045)	29
Brennan Island	Not measured					0.038	0.125	0.072	0.069 (0.010)	8
Saranac Island	0.384	2.265	0.498	0.677 (0.118)	16	0.058	6.704	0.437	0.620 (0.156)	41

**Table 2:** Okadaic acid in discrete water samples and SPATT.

Site	Water Okadaic Acid Concentration (ng OA/L)					Okadaic Acid Concentration in SPATT (ng OA/g SPATT Dry Weight/Day)				
	Min	Max	Median	Mean (SE)	n	Min	Max	Median	Mean (SE)	n
Ahlstrom	14.86	24.96	19.89	20.16 (1.36)	7	2.13	33.32	13.50	13.04 (2.95)	11
Concepcion	12.41	44.56	23.18	25.16 (3.50)	10	3.63	56.06	23.11	21.81 (3.78)	13
Millar	19.65	24.70	22.17	22.17 (2.53)	2	5.32	35.54	11.85	15.06 (2.41)	16
Raza Island	17.29	29.50	23.65	23.68 (1.75)	6	4.03	34.26	19.61	17.20 (3.24)	10
Brennan Island	Not measured					1.09	7.69	3.88	3.70 (0.82)	7
Saranac Island	12.70	38.16	21.45	24.06 (3.48)	7	14.26	36.81	20.35	23.37 (2.52)	10

**Table 3:** Domoic acid (DA) in SPATT.

Site	Domoic Acid Concentration in SPATT (pg DA/g SPATT Dry Weight/Day)				
	Min	Max	Median	Mean (SE)	n
Saranac Island	27.1	145.5	70.5	75.2 (11.6)	9
Concepcion	5.9	43.0	20.93	22.7 (3.2)	12
Millar	9.1	42.3	16.4	20.7 (3.6)	10
Raza Island	5.1	2223.0	52.1	473.6 (437.4)	5
Ahlstrom	7.6	102.1	17.1	29.0 (12.4)	7

**Table 4:** Dinophysistoxin-1 (DTX-1) in SPATT.

Site	DTX-1 Concentration in SPATT (ng DTX-1/g SPATT Dry Weight/Day)				
	Min	Max	Median	Mean (SE)	n
Saranac Island	15.4	36.1	18.5	22.3 (3.3)	7
Millar	3.1	31.1	10.4	13.8 (12.2)	4
Raza Island	2.8	55.6	43.9	37.2 (20.8)	5
Ahlstrom	5.9	205.8	24.5	65.2 (47.1)	4

**Table 5:** Pectenotoxin-2 (PTX-2) in SPATT.

Site	PTX-2 Concentration in SPATT (ng PTX-2/g SPATT Dry Weight/Day)				
	Min	Max	Median	Mean (SE)	n
Saranac Island	34.2	58.6	38.6	40.8 (3.1)	7
Millar	4.1	26.4	15.9	15.6 (5.0)	4
Raza Island	9.4	97.5	65.9	57.2 (14.7)	5
Ahlstrom	10.9	142.3	48.2	62.4 (30.2)	4

**Table 6:** Yessotoxin (YTX) in SPATT FIX.

Site	YTX Concentration in SPATT (ng YTX/g SPATT Dry Weight/Day)				
	Min	Max	Median	Mean (SE)	n
Saranac Island	3.1	729.7	55.9	191.4 (104.1)	7
Millar	9.4	39.0	27.7	25.4 (8.6)	3
Raza Island	4.6	72.3	20.2	29.3 (15.5)	4
Ahlstrom	9.4	144.9	31.7	62.0 (42.0)	3

**Table 7:** Cumulative toxin (ng toxin) uptake for a 1 kg fish based on the drinking rate for Atlantic Salmon in seawater (0.01 L/h/kg) (Laronde and Brauner, unpublished). Water toxin concentrations used were the minimum, median, and maximum values for microcystin and okadaic acid (ng toxin/L water) measured across all sites.

		Water Toxin Conc.	Duration of Drinking				
		(ng/L)	Hour	Day	Week	Month	Year
	Min	0.1	0.00	0.04	0.25	0.99	11.91
Microcystin	Median	0.4	0.00	0.10	0.73	2.93	35.16
	Max	2.3	0.02	0.54	3.81	15.22	182.65
	Min	12	0.1	3.0	20.8	83.4	1000.7
Okadaic acid	Median	22	0.2	5.2	36.5	146.1	1753.1
	Max	45	0.4	10.7	74.9	299.4	3593.3

**Table 8:** Environmental data for the sites and time period where measurements are available.

Site	Date Range ^1^	Parameter	Min	Max	Median	Mean (SE)	n
Ahlstrom	1 June 2017–27 June 2019	Dissolved O_2_ (mg/L)	5.3	15.2	8.8	8.8 (0.1)	595
		Salinity (psu)	15.0	33.5	27.5	26.9 (0.1)	
		Temperature (°C)	6.7	20.6	13.5	12.9 (0.2)	
Concepcion	1 June 2017–26 June 2019	Dissolved O_2_ (mg/L)	41.1	26.5	8.9	8.9 (0.1)	630
		Salinity (psu)	9.0	27.5	27.5	26.4 (0.2)	
		Temperature (°C)	4.5	16.2	10.0	10.6 (0.1)	
Dixon Bay	9 February 2018–7 June 2019	Dissolved O_2_ (mg/L)	6.0	10.8	8.8	8.6 (0.1)	307
		Salinity (psu)	16.0	33.0	29.0	28.1 (0.2)	
		Temperature (°C)	7.7	13.7	10.4	10.5 (0.1)	
Millar	28 September 2017–7 June 2019	Dissolved O_2_ (mg/L)	6.4	11.5	8.9	8.7 (0.1)	282
		Salinity (psu)	16.5	33.0	29.5	28.6 (0.1)	
		Temperature (°C)	7.9	15.7	10.1	10.6 (0.1)	
Raza Island	10 October 2017–7 June 2019	Dissolved O_2_ (mg/L)	5.0	12.2	8.4	8.5 (0.0)	621
		Salinity (psu)	19.5	31.0	28.0	27.5 (0.1)	
		Temperature (°C)	6.4	18.5	9.5	10.1 (0.1)	
Saranac Island	28 February 2019–7 June 2019	Dissolved O_2_ (mg/L)	8.5	11.8	9.4	9.5 (0.0)	129
		Salinity (psu)	26.0	29.0	26.0	29.2 (0.1)	
		Temperature (°C)	7.5	13.9	10.8	10.9 (0.2)	

^1^ date in day month year.

**Table 9:** Parameters for LC–MS/MS analysis of biotoxins in SPATT extracts.

Analyte	Retention	ESI	Primary Transition (*m*/*z*) *	Collision Energy (V)		Secondary	Collision
	Time (min)	Mode				Transition *m*/*z*	Energy (V)
DTX-1	9.39	(-)	817.0 > 254.7		40	817.0 > 113.0	40
PTX-2	8.26	(+)	876.3 > 823.0		30	876.3 > 213.0	30
YTX	8.53	(-)	1141 > 1061		46	1141 > 855.0	60

* *m*/*z* = mass-to-charge ratio.

## Data Availability

Datasets are available on request: the raw data supporting the conclusions of this article will be made available by the authors, without undue reservation.
